# Mouse Models to Examine Differentiated Thyroid Cancer Pathogenesis: Recent Updates

**DOI:** 10.3390/ijms241311138

**Published:** 2023-07-06

**Authors:** Hye Ryeon Choi, Kwangsoon Kim

**Affiliations:** 1 Department of Surgery, Eulji Medical Center, Eulji University School of Medicine, Seoul 01830, Republic of Korea; 2bethere@naver.com; 2 Department of Surgery, College of Medicine, Catholic University of Korea, Seoul 06591, Republic of Korea

**Keywords:** thyroid cancer, papillary, follicular, differentiated, mouse model, pathogenesis

## Abstract

Although the overall prognosis of differentiated thyroid cancer (DTC), the most common endocrine malignancy, is favorable, a subset of patients exhibits aggressive features. Therefore, preclinical models that can be utilized to investigate DTC pathogenesis and novel treatments are necessary. Various mouse models have been developed based on advances in thyroid cancer genetics. This review focuses on recent progress in mouse models that have been developed to elucidate the molecular pathogenesis of DTC.

## 1. Introduction

The incidence of thyroid cancer, the most common endocrine malignancy [1], has been increasing in the USA over the last several decades [2]. Especially in South Korea, the incidence rate of thyroid cancer in 2011 was 15 times higher than in 1993 due to the increased detection of papillary thyroid cancer [3]. In fact, data from the Surveillance, Epidemiology, and End Results-18 cancer registry (SEER) indicate substantially increased incidence-based mortality rates in patients with thyroid cancer [4]. Follicular thyroid cells and parafollicular C cells are the two main components of the thyroid gland [5]. Differentiated thyroid cancer (DTC), which arises from follicular cells, is the most common form of thyroid cancer and includes papillary thyroid carcinoma (PTC) and follicular thyroid carcinoma (FTC), which account for 80% and 6–7% of all thyroid cancers, respectively. Nowadays, Hurthle cell carcinoma (HCC), which accounts for 3–4% of all thyroid cancers, is considered a unique type of DTC, distinct from FTC [6]. Poorly differentiated thyroid cancer (PDTC) and anaplastic thyroid cancer (ATC) also arise from follicular thyroid cells, representing approximately 5–6% of all thyroid cancers [7]. On the other hand, medullary thyroid carcinoma (MTC) arises from parafollicular C cells, which makes up 2–3% of all thyroid cancers [8,9,10].

Although the vast majority of patients with DTC have a favorable prognosis, with 5-year overall survival of more than 95%, a subset of patients shows aggressive features. Approximately 2% of patients have distant metastasis at initial diagnosis [9], and locoregional recurrence is reported in up to 20% of patients [11]. The mainstay of treatment in metastatic DTC is surgical thyroidectomy followed by adjuvant radioactive iodine ablation. However, DTC responds poorly to systemic therapy, and radioactive iodine ablation is often not curative. Between 7% and 23% of patients develop distant metastases, with approximately two-thirds of these individuals becoming unresponsive to radioactive iodine treatment [12]. Therefore, the prognosis of metastatic thyroid cancer is markedly diminished. The role of external beam radiation therapy (EBRT) in patients with metastatic DTC is controversial, although few groups reported the efficacy of EBRT for selected locally advanced or recurrent non-anaplastic thyroid cancer [13,14]. The latest American thyroid association management guidelines stipulated that there is no role for routine systemic adjuvant therapy in patients with DTC [13]. While the recent introduction of tyrosine kinase inhibitors has been associated with notable prognostic improvement in radioiodine-refractory thyroid cancer, the response time is usually limited [15]. Therefore, translational research on the pathogenesis of thyroid cancer is emerging for the development of novel therapeutics.

There are many established human thyroid cancer cell line models for use in in vitro tissue culture systems or in in vivo xenograft studies. The currently available and widely used genetically engineered mouse models (GEMs) not only provide information on the molecular pathogenesis of thyroid cancer but also facilitate the preclinical testing of new therapeutics [8,16]. This review focuses on recent advances made in the understanding of the molecular pathogenesis of DTC using mouse models.

## 2. Mouse Models of DTC

### 2.1. Mouse Models

Thyroid cancer cell lines derived from human tumors are widely used to investigate the mechanisms of cancer initiation and progression and to test new chemotherapeutics. Research on thyroid cancer can be conducted with cell culture in vitro or immunosuppressed xenograft models in vivo. Mouse models are often used to study a wide range of cancers as they provide several advantages over other animal models, including short life span, small body size, and genetic and molecular similarities to humans [17,18]. Traditional experimental paradigms include subcutaneous implantation of human cancer cell lines to immunocompromised mice [19]. Usually, athymic nude mice with severe combined immunodeficiency and NOD.Cg-Prkdcscid Il2rgtm1Wjl/SzJ mice are used as immunocompromised models [20,21]. Orthotopic implants of thyroid cancer cells introduced into the thyroid bed were used to develop a more physiologic environment for carcinogenesis in a recently described approach [22], whereas thyroid cancer cells were injected into the tail vein or cardiac vessels to recapitulate metastatic settings [20,23]. Although these models are useful, cancer models in immune-competent hosts are needed. To that end, genetically engineered mouse models using the Cre-loxP system have garnered increased attention in recent years [24,25]. Advanced genetic recombination has enabled the creation of novel mouse models of thyroid cancer that can reproduce complicated clinical scenarios and tumor heterogeneity [16]. In this section, we are going to discuss the most common mouse models.

#### 2.1.1. Cell Line-Derived Xenograft Models

Cell line-derived xenograft models (CDXMs) are created by implanting human cancer cell lines into immunodeficient mice. Authenticated cell lines such as 8505C, PTC-1, and FTC 133 are widely used to establish CDXMs by inoculating Matrigel-suspended thyroid cancer cells into immunodeficient mice [16].

Cross-contamination and misidentification of cell lines are critical and common concerns that can negatively impact the reliability of published studies [26,27,28]; therefore, choosing proper cell lines and verifying the findings are critical. Indeed, several studies were conducted to validate the integrity of human thyroid cancer cell lines [29,30]. The number of cell lines derived from thyroid cancer is considerably smaller when compared to other common cancers, and their characteristics have not been well studied [29]. Out of the 1100 specimens evaluated by the Cancer Cell Line Encyclopedia, there are only 18 thyroid cancer cell lines. Additionally, some of these cell lines may be repetitive or have uncertain origins [29,31,32].

Recent advances in next-generation sequencing technology have allowed the authentication and characterization of the genomic landscape of cell lines. Next-generation sequencing (NGS) is useful to evaluate whether cell lines properly replicate the characteristics of the original cancer and whether specific traits become more prominent during cell culture in the laboratory [28,29,30,33,34]. Landa et al. performed NGS and analyzed the 60 established thyroid cancer cell lines [29]. They found that all thyroid cancer cell lines exhibited a significant depletion of thyroid differentiation markers, regardless of their origin. 

According to the site of implantation, CDXMs are classified into subcutaneous, orthotopic, and metastatic models. The injection sites of cancer cell lines are the right flank, thyroid bed, and tail vein or intracardiac, respectively [35]. Mycoplasma-free thyroid cancer cells are harvested and counted using an automated cell counting system. Afterwards, the cells are resuspended with phosphate-buffered saline (PBS). With the aid of a microscope, an equal amount of cancer cells suspended in PBS are injected into sterilized mice [35]. Morrison et al. created multiple orthotopic models using different thyroid cancer cell lines. They observed variations in take rates. The cell lines 8505C, T238, K1/GLAG-66, and BCPAP showed >90% take rates in the orthotopic model [35]. While monitoring cancer progression and treatment response are easily achieved in subcutaneous CDXMs, these models cannot mimic the tumor microenvironment and metastatic patterns [36]. Numerous studies have reported that the phenotype of cancer cells can be altered through interaction with neighboring tissues. The failure to recapitulate the tumor microenvironment is a major drawback of subcutaneous CDXMs [20,37]. While orthotopic implantation of cancer cells can recapitulate the local invasion and metastasis of DTC and can stimulate the microenvironment [25], its dependence on athymic nude mice, which lack normal immune function, is a significant limitation. Consequently, this model does not completely represent human disease, and evaluating the role of the immune system in DTC progression is not possible [38]. Several mouse models of metastatic cancer have been demonstrated to reliably reproduce the metastatic patterns of thyroid cancer observed in human patients [21]. However, these models have several limitations. First, the rapid expansion of the tumor does not allow enough time for an adaptive immune response [39]. Additionally, the implantation of cancer cells through a wound may not precisely imitate the local invasion of human thyroid cancer, which is a challenge in the evaluation of extrathyroidal extension [40].

#### 2.1.2. Patient-Derived Xenograft Models

Patient-derived xenograft models (PDXMs) use patient-derived tissue or cancer cells for transplantation. Fresh thyroid tumor is surgically obtained from patients. Cell lines are created by generating single cells through enzymatic digestion and culturing the collected single cells [21]. The process of developing PDXMs is the same as that of CDXMs. Maniakas et al. developed subcutaneous PDX models using six ATC tumors. The PDX models underwent mutation analysis and histopathologic characterization, demonstrating a high degree of similarity and fidelity to the original tumors [21].

Growth of the transplanted cancer tissue generates a tumor environment, which is valuable for investigating cancer pathogenesis and heterogeneity as well as drug efficacy and safety [21,41]. Multiple PDXMs have been established for a wide range of cancers, including lung, ovary, pancreas, and breast cancer; however, PDXMs for thyroid cancer are limited [42,43,44,45]. PDXMs enable real-time testing of novel chemotherapeutics. Several preclinical trials used PDXMs to evaluate targeted therapy for thyroid cancer, including vemurafenib for PTC, the selective RET kinase inhibitor LOXO-292 for *RET*-mutated thyroid cancer, PLX51107 and PD0325901 for ATC, and cabozantinib for MTC [46,47,48,49]. PDXMs are expected to precisely reflect the individual molecular pathogenesis of thyroid cancer, which is essential for the development of personalized therapeutics.

#### 2.1.3. Syngeneic Models

Syngeneic models are established by engrafting mouse cancer cells or tissues into immunocompetent mice with the same genetic background. Vanden Borre et al. generated a syngeneic ATC orthotopic mouse model using murine thyroid cancer cell lines. The tumor cell lines were transduced with lentiviral vectors and implanted in the thyroid glands of syngeneic mice. Rapid and synchronous tumor formation was detected within one week of implantation, with immune cell infiltration [40].

These models are useful in assessing cancer immunology and the effect of immunotherapy. Several studies on immunocompetent orthotopic mouse models of ATC, PTC, and FTC have reported rapid tumor expansion with significant immune cell infiltration of the tumor [40,50,51]. Although the short latency of tumor development is an advantage, the rapid tumor growth does not allow enough time for immune editing or the development of distant metastases [40,52].

#### 2.1.4. GEMs

GEMs are established by altering the genome and are, therefore, distinct from the mouse models that utilize grafts [53]. In this approach, transgenic mice are usually created by injecting functional DNA pieces into fertilized oocytes [54]. Recent advances in designer nucleases, such as transcription activator-like effector nucleases, zinc-finger nucleases, and the clustered regularly interspaces short palindromic repeats (CRISPR)/CRISPR-associated (Cas) 9 system, have made it feasible to perform rapid, targeted genetic modifications [55]. Currently, the Cre-loxP system is widely used for gene editing [56]. Desired alterations ranging from targeted point mutations to large site-specific chromosomal aberrations can be achieved in the mouse genome [57]. Moreover, conditional transgenesis or conditional knockouts can be created through the use of recombinases [57,58]. Promoters specific to thyroid tissue, such as thyroglobulin (Tg), thyroid peroxidase (TPO), and calcitonin, can be used to temporally or spatially control oncogene expression [59,60,61]. Tg-CreER^T2^/Braf^CA^ and TPO-CreER^T2^/Braf^CA/+^ are known GEMs for PTC [62,63]. GEMs allow the gradual development of tumors and are suitable for investigating interactions between tumor cells, tumor microenvironment, and the immune system [64]. However, the stroma and immune cells that interact with the xenogeneic tumor are of mouse origin and may be different from those in patients with thyroid cancer [65]. Additionally, the long latency to tumorigenesis and high cost are challenging issues of GEMs [39].

The characteristics, advantages, and drawbacks of mouse models of DTC are summarized in Table 1.

### 2.2. GEMs of DTC

The discovery of genetic and molecular alterations using mouse models of thyroid cancer has expanded our knowledge of thyroid cancer pathogenesis, dedifferentiation, proliferation, and metastasis. These studies demonstrated the involvement of the RTK, RAS/RAF/MEK/ERK, PI3K/AKT/mTOR, SRC, and JAK-STAT signaling pathways in thyroid cancer pathogenesis. Through studying mouse models of thyroid cancer, it has been determined that the RTK signaling pathway plays a crucial role in regulating angiogenesis, proliferation, and metastasis [66]. In general, the presence of *RAS* mutations is closely linked to the progression of thyroid tumors. The combined occurrence of *BRAF* mutations and *RAS* mutations may play a role in the formation and advancement of thyroid cancer. Various forms of RAS (H-, K-, and N-RAS) and their downstream targets facilitate the migration of thyroid tumor cells [67,68]. The signaling pathway known as PI3K/AKT/mTOR plays a role in promoting the proliferation of thyroid cancer cells and contributes to their survival and angiogenesis processes [69]. SRC family kinases play a role in regulating important cellular processes, including cell proliferation, motility, invasion, and angiogenesis [70]. JAK-STAT3 pathway potentially plays a role in the growth, progression, and spreading of thyroid tumors [71].

Activation of the RAS/RAF/MEK/ERK, i.e., mitogen-activated protein kinase (MAPK), signaling pathway facilitates the malignant transformation of follicular cells to PTC [72]. *RET/PTC* and *NTRK* rearrangements and *RAS* and *BRAF* mutations are common activating mutations in the MAPK pathway. The upregulation of the PI3K/AKT/mTOR pathway can induce the malignant transformation of follicular cells to FTC. FTC is related to activating mutations in *RAS, PIK3CA*, and *AKT1* genes and to the inactivation of PTEN. On the other hand, dysfunction of the RTK pathway is a more commonly observed finding in MTC. The activation of the WNT/β-catenin pathway is related to the progression and dedifferentiation of thyroid cancer, whereas inactivating mutations in *TP53* and activating mutations in the *TERT* promotor are widely reported in aggressive and undifferentiated thyroid cancer [16,67,73,74,75]. The remainder of this review will focus on GEMs of PTC and FTC.

#### 2.2.1. PTC

Activation of the MAPK signaling pathway is a hallmark of PTC. The V600E mutation of *BRAF*, one of the components of the MAPK signaling pathway, is the most common driving mutation in sporadic PTC and is found in 40–60% of all PTCs [72,76]. On the other hand, *RET*/*PTC* gene fusions account for 15–20% of all PTCs and are mainly related to PTC in pediatric patients and those with radiation exposure [76]. The most frequently detected RET fusion genes are *RET/PTC1* and *RET/PTC3* [77]. Rearrangement of *NTRK1* is also coupled to the MAPK signaling pathway, with a much lower incidence compared to those of the *RET*/*PTC* gene fusions. *RAS* oncogenes are responsible for 10% of all PTCs and are common in patients with the follicular variant of PTC [78].

##### Models of *Braf*-Driven GEMs

The *BRAF* V600E mutations are associated with aggressive clinicopathologic behavior and poor prognosis in PTC [79]. The first *Braf*-driven GEM used the bovine Tg promoter with *Braf* V600E cDNA overexpression in the thyroid cells of transgenic FVB/N mice in a study that used two distinct lines, Tg-*Braf2* and Tg-*Braf3*. These mice with thyroid-specific *Braf* V600E expression developed PTC with characteristics similar to those of *BRAF*-positive human PTC. The penetration rate was high, and PTC was detected in >90% of the mice by 12 weeks. Most of the cancers arising in these models were tall-cell variants of PTC with locally invasive features and no distant metastasis. However, the expression of *Braf* transgene resulted in the dedifferentiation of cells, which lost thyroglobulin (Tg) expression and eventually tended to lose the Tg-driven transgene expression [80]. This closely resembles the phenotype observed in humans with *BRAF* V600E-positive PTCs, providing strong evidence for the oncogene’s significant role in the development of this cancer. Consequently, this animal model is expected to be an invaluable tool for gaining a deeper understanding of the molecular events involved in the dedifferentiation of PTC. Furthermore, this model offers a valuable platform for testing potential therapeutic strategies for the treatment of PTC, as well as interventions aimed at preventing tumor dedifferentiation [80].

One study used the conditional activation of *Braf* V600E to overcome this caveat. In mice harboring tamoxifen-inducible Cre (CreERT2) under the control of the Tg promoter and the Cre-activating *Braf* V600E, PTC was observed six months after the injection of tamoxifen [62]. Another group developed doxycycline-inducible thyroid expression in a *Braf* V600E mouse model. The authors confirmed the formation of highly penetrant and poorly differentiated thyroid cancer after one week of doxycycline exposure and observed that the discontinuation of doxycycline reversed the malignant phenotype into normal follicular tissue [81].

In these GEMs, one common issue is that the expression of the *Braf* transgene leads to the dedifferentiation of follicular cells, which results in thyroid hypofunction with elevated thyroid-stimulating hormone (TSH) levels. Since the Tg promoter is also regulated by TSH, the higher TSH level induces oncogene expression and complicates the analysis of Tg-*Braf* mouse lines [80]. As an alternative approach, TPO-driven Cre was used to activate *Braf*. The authors reported that knockout of the TSH receptor impaired *Braf*-induced PTC development [82]. In another TPO-Cre model, McFadden et al. reported that the *Braf* V600E mutation initiated PTC and that the additional loss of *Tp53* constrained the progression to ATC. Although *Braf* inhibition alone did not completely suppress PTC progression to ATC, the combined inhibition of *Mek* and *Braf* effectively controlled ATC progression [63,83]. One study using another mouse model demonstrated that the combined knockout of *Tp53* and *Pten* resulted in ATC [83]. Additional activation of *Pik3ca* or suppression of *Pten* was also related to the progression of Braf-induced PTC to ATC [84].

Several studies have reported a distinct correlation between *BRAF* V600E and molecular characteristics, indicating both biological and clinical aggressiveness in humans [85,86]. On the other hand, Xing et al. reported an independent association between *BRAF* mutation and the recurrence of PTC in their study, including 2099 patients [87]. Consequently, the present role of mutated *BRAF* in risk stratification for PTC remains restricted. Enhancing our comprehension of *BRAF* mutations has the potential to enhance the utility of the mutation in the prognostic applications [88].

##### Models of RTK Fusion Proteins

*RET*/*PTC* and *NTRK* rearrangements are known drivers of PTC [77,89]. Few studies utilized *RET*/*PTC1* and *RET*/*PTC3* transgenic mouse models [90,91,92,93,94]. In a transgenic mouse line expressing the *RET*/*PTC1* oncogene using the rat Tg promoter, Santoro et al. reported a long latency period of PTC development by 16 months and no distant metastasis [94]. Other mouse models with the thyroid-targeted expression of the *RET*/*PTC1* oncogene under the control of the bovine promoter were reported by Jhiang et al., 1996 [90]. *Ret*/*Ptc1* mice with high copy numbers of the transgene developed PTC as early as 18 embryonic days [93]. Furthermore, the additional loss of p53 function led to anaplasia and metastatic cancer in *Ret/Ptc1* transgenic mice. The authors concluded that the absence of a functional p53 protein, while not sufficient for the development of a malignant phenotype, aided other oncogenes in promoting tumorigenesis [95]. Buckwalter et al. investigated the contribution of signal transduction pathways mediated by the phosphotyrosine 294, 404, and 451 residues of PTC1 to the formation of *Ret*/*Ptc1*-induced thyroid cancer by generating Tg-TPC1 transgenic mice carrying key tyrosine site mutations. The rate of tumor formation was 30% lower in mice carrying these tyrosine site mutations than in those with *Ret/Ptc1* not harboring these mutations, which was a significant difference. The authors argued that these signaling pathways played an important role in transformation but that none of them were exclusively necessary for tumorigenesis [96]. Similar to the *Ret*/*Ptc1* mouse model, mice with transgenic *Ret*/*Ptc3* driven by the bovine Tg promoter developed tumors resembling the solid variant of PTC in humans; approximately one-third of the animals exhibited axillary lymph node metastasis [92]. The closest genetic similarities between murine tumors and human PTC were found in 2-month-old mice. Since *Ret*/*Ptc* rearrangements were in the germline in mice, in contrast to the sporadic genetic alterations that lead to these rearrangements in humans, PTC development and progression might be more aggressive in mice compared to humans [97]. The rearrangement of *NTRK1* results in the overactivation of the MAPK pathway [98,99]. About half of the transgenic mice expressing the TRK-T1 fusion protein develop follicular hyperplasia and PTC [100]. Further, additional ablation of the *p27* tumor suppressor gene increased PTC prevalence [100].

##### TSH Receptor and Cyclic Adenosine Monophosphate Signaling Pathways

TSH receptor (TSHR) is a glycoprotein hormone receptor that binds TSH and plays a pivotal role in cyclic adenosine monophosphate (cAMP) generation and the subsequent activation of protein kinase A (PKA) signaling [98]. The TSHR/cAMP/PKA pathway is an important foundation for TSH suppression therapy in patients with thyroid cancer. However, the role of this pathway in the initiation of thyroid carcinoma is unclear. Although abnormalities in the PKA signaling pathway are associated with McCune–Albright syndrome, which is characterized by endocrine hyperplasia/hyperfunction, *PKA* mutation is uncommon in thyroid tumors [101]. Since the adenosine A2 receptor can also activate the cAMP cascade, Ledent et al. generated a transgenic mouse line expressing the canine A2 adenosine receptor under the control of the bovine Tg promoter and reported that these mice developed severe hyperthyroidism with goiter but not thyroid cancer [102,103]. These results suggest that *Tshr* or *Pka* overactivation alone is not sufficient to initiate tumorigenesis but does accelerate the progression of existing PTC in mice.

#### 2.2.2. FTC

About 45% of FTCs exhibit single-point mutations in genes of the *RAS* family, with *N-RAS* activating mutations at codon 61 being the most commonly observed mutation [104]. Gene fusion of *PAX8* and *PPARG* to create the PPFP fusion protein is another common genetic alteration found in approximately 35% of FTCs [105]. FTC with *PAX8/PPARG* rearrangement is likely to occur in younger patients and commonly exhibits vascular invasion [105,106,107,108]. Other infrequent mutations in FTC are amplification or mutation of *PIK3CA* and mutation of *PTEN* [76,109]. One of the two inherited syndromes related to FTC is Cowden syndrome, which is caused by germline *PTEN* and *SDHx* mutations. Standardized incidence rates for all kinds of thyroid cancer were 72, and FTC was overrepresented in patients with *PTEN* mutations compared to those with *SDHx* mutations [110]. Carney complex is another autosomal-dominant tumor syndrome caused by mutations in *PRKAR1A*, which acts as a tumor suppressor; 2.5% of individuals with Carney complex develop thyroid cancer [101,111].

##### Models of RAS Activation

*RAS* mutation is a well-known driver of FTC. *RAS* activations are found in nearly 50% of all conventional FTCs [112,113]. *RAS* mutations are closely associated with the follicular structure of DTC. It is not only found in FTC but also found in 10-20% of PTC, which is almost always a follicular variant [114]. *RAS* mutations are also reported in adenomas and hyperplastic benign nodules [115]. Puzziello et al. reported that benign thyroid nodules containing *RAS* mutations tend to show accelerated growth [116]. On the other hand, in the review of 2021, the authors concluded that the impact of *RAS* mutations on both normal and transformed thyroid cells, as well as their impact on clinical characteristics of thyroid neoplasms, should not be considered by itself. Instead, they should be considered in conjunction with other genetic abnormalities within a more intricate context [117].

However, thyroid cancer does not develop in several *RAS* activation models, including those in transgenic or Cre-activated knockin mouse lines. [103,118,119,120,121]. Mice with the thyroid-specific expression of activated *K-ras* only develop mild follicular proliferation. In one study, only one case developed FTC without local or distant invasion after stimulation with a goitrogen for six months [118]. In another study, mice with the endogenous expression of *H-ras* G12V did not develop thyroid tumors [121]. *K-ras* or *H-ras* mutations alone are not sufficient for the development of thyroid cancer. On the other hand, the incidence of FTC was 40% in a study using *N-ras* transgenic mice, and dedifferentiation with distant metastasis was observed in 25% of the animals [122]. *N-RAS* mutations are linked to the dedifferentiation of tumor cells and unfavorable prognosis in human thyroid cancer [123]. The cell line model demonstrated that the aggressive biologic characteristics of FTC with *N-Ras* mutations were due to chromosomal instability induced by the *Ras* mutation [124]. These findings suggest that secondary mutations might account for tumorigenesis in FTC. However, one of the drawbacks of these transgenic mouse models is that the cancer phenotype produced by the overexpression of mutated *Ras* may not accurately represent the activity of the endogenous mutant *Ras* expressed at physiologic levels [125].

Thus, Miller et al. developed a mouse model that expressed *K-ras* G12D under the control of the endogenous *K-Ras* promoter, which regulated the expression of the oncogenic allele at the endogenous level. Similar to that observed in other models, the constitutive activation of *K-Ras* alone was not sufficient to transform the thyroid cells. The authors crossed this mouse line with another line harboring *Pten* deletion targeted to the thyroid epithelium and found that the simultaneous *K-Ras* activation and *Pten* deletion were highly oncogenic. In addition, all dual-mutant mice developed aggressive, invasive FTC, where half of the mice died within 7 weeks after birth, and all mice that survived longer than 12 weeks developed lung metastases [120].

The existing data slightly indicate an adverse prognostic impact associated with mutated *RAS* in human DTC, including higher rates of distant metastases, recurrence, and mortality [126,127]. The most possible theory is that the mutations causing aberrant *RAS* activation may trigger a progression from well-differentiated cancers to less differentiated forms [107]. Therefore, more mouse model studies that specifically focus on *Ras* mutants are necessary for a better understanding of the prognostic significance of *RAS* mutations in FTC and DTC.

##### Models of Rap1b

Rap1, a small G protein, belongs to the RAS family and uses a cAMP-dependent mechanism to transmit signals from the TSHR to the MAPK pathway [128]. Rap1 is suggested to play an important role in thyroid tumorigenesis. Rap1b can either stimulate or inhibit cell proliferation, with its effects depending on the cell-specific signal transduction programs [129]. Ribeiro et al. developed a transgenic mouse line that expressed constitutively active Rap1b G12V [129]. The model was designed to toggle G protein activity using a switch, and the stimulation of Cre activity by tamoxifen stopped the expression of the active Rap1b G12V protein, leading to the production of the inactive Rap1b S17N mutant. Similar to that observed in *Ras* mutant models, these mice did not exhibit a harmful phenotype. Exposure of the Rap1b G12V mice to a goitrogenic protocol for six months resulted in nodular thyroid hyperplasia, which was completely reversed after goitrogen removal. When Rap1b G12V was switched to Rap1b S17N with tamoxifen treatment, an approximately 50% decrease in thyroid gland size was noted despite continued goitrogen treatment, which indicated that Rap1-GTP was required to maintain the hyperplastic state. After 12 months of goitrogen treatment, some mice with the active Rap1b G12V protein developed locally invasive FTC. Altogether, these findings indicate that the oncogenic effect of Rap1b G12V is linked to the induction of TSH-mediated signaling.

##### Models of PAX8/PPARG Fusion

*PAX8* is a crucial regulator of thyroid development and induces the expression of thyroid-specific genes encoding thyroglobulin, TPO, and the sodium iodide symporter [130]. Although approximately 35% of FTCs are associated with *PAX8/PPARG* fusion, the role of this genetic alteration in FTC development is poorly understood. Transgenic mice expressing the PAX8/PPARG fusion protein (PPFP) developed thyroid hyperplasia but not carcinoma. The PPFP expression also had a synergistic effect with *Pten* deletion to cause marked thyroid hyperplasia but not FTC, indicating that additional events were necessary for the development of FTC [131]. On the other hand, knocking out *Pten* in PPFP models succeeded in the development of aggressive FTC with vascular/perithyroidal invasion and lung metastasis. Interestingly, the administration of the PPARG agonist pioglitazone resulted in a significant reduction in tumor growth, with the cells exhibiting signs of development into adipocytes instead. Significantly, this results in a distinctive antitumor response and suggests that pioglitazone treatment could be effective for patients with PPFP thyroid carcinomas [132].

##### *Pten* Knockout Models

Germline mutations in the tumor suppressor gene *PTEN* can cause Cowden syndrome, characterized by hamartomas in multiple organs and increased risk of breast, thyroid, and other cancers [133]. *Pten* is essential for embryonic development in mice, as complete *Pten* knockout leads to embryonic lethality [134]. On the other hand, thyrocyte-specific silencing of *Pten* in mice led to contradictory results. Diallo-Krou et al. reported the development of only follicular adenoma in these thyroid-specific knockout mice [135]. However, a subsequent study suggested that the activation of PI3K via *Pten* deletion was sufficient to drive metastatic FTC [136]. Tiozzo et al. hypothesized that the conflicting results of these studies might reflect differences in the genetic background across these mouse strains [137].

##### *Prkar1a* Knockout Models

The inactivation of *Prkar1a*, which is the regulatory subunit of the cAMP-dependent PKA, is known to result in increased PKA activity [111]. In conventional *Prkar1a* heterozygous mouse models, about 11% of mice older than 13.5 months of age developed malignant thyroid neoplasms [138]. In a subsequent study, Pringle et al. reported that over 40% of the mice developed locally advanced FTC in a model of tissue-specific *Prkar1a* knockout [139]. Dual-hit models for FTC have also been generated by the same groups. In one study, the double knockout of *Prkar1a* and *Pten* led to aggressive FTC with 100% penetrance by 8 weeks of age. Furthermore, well-differentiated lung metastasis was detected in 27% of the mice [140].

##### Models of Thyroid Hormone Receptor β PV Variant

Knockin mouse models of the thyroid hormone β PV variant (*TRβ-PV*) were originally developed to model the inherited resistance to thyroid hormone. Notably, homozygous *TRβ-PV* mice develop FTC as they become older, and the disease progression follows a pattern similar to that observed in human FTC [141]. These mice develop invasive FTC at 4–5 months of age, and distant lung and heart metastases are observed after 5 months of age [141]. This model has been widely used to investigate the patterns of progression and genetic alterations in FTC. Kato et al. crossbred *TRβ-PV* mice with *TRβ* knockout mice and observed spontaneous development of FTC with lung metastasis in *TRβ-PV*/- heterozygous mice, suggesting that the presence of a single mutated *TRβ* allele was sufficient for the spontaneous development of FTC. The authors suggested the possibility that *TRβ* could function as a tumor suppressor and might be considered a novel therapeutic target [142]. Guigon et al. generated *TRβ (PV/PV) Pten (+/−)* mice to investigate the role of *Pten* in carcinogenesis. The authors demonstrated that the deletion of *Pten* accelerated the progression of thyroid cancer and increased lung metastasis, leading to a marked reduction in survival rate [143]. The association between the activation of the PI3K/AKT pathway and FTC is well established [144]. The activation of this pathway was also identified in metastatic lesions of *TRβ-PV* mice [145], and treatment with LY294002, a potent PI3K inhibitor, led to decreased tumor growth and increased survival in these mice [146]. Saji et al. crossed *Akt1* knockout mice with *TRβ* knockin mice; the resulting *TRβ-PV/PV-Akt1* knockout mice exhibited delayed development of thyroid cancer with a less invasive phenotype, which indicated that the development and progression of thyroid cancer were *Akt1*-dependent in this mouse model [147]. Numerous studies have been conducted to clarify the mechanisms of FTC carcinogenesis and metastasis using *TRβ-PV* knockin mouse models [148,149,150,151].

##### Alpha 1b-Adrenergic Receptor Mutant Model

A model for FTC was established using the bovine Tg promoter to control mutated alpha 1b-adrenergic receptor gene expression. Induction of the promoter activity resulted in the constitutive activation of the TSH signaling pathway. In this model, the mice started developing goiter in the first few weeks of life and thyroid nodules with increasing age; some mice developed locally invasive FTC. Lung metastases were observed in approximately 20% of the mice older than 12 months [152].

## 3. Methods

Publications were collected from the PubMed database. The last search was on 31 December 2022. The following keywords were used for the search: “mouse model, DTC (229 results)”, “mouse model, PTC, pathogenesis (209 results), and “mouse model, FTC, pathogenesis (85 results)”. Eligibility criteria included results published after 1 January 2011.

Articles on mouse models to study the molecular pathogenesis of differentiated thyroid cancer were included. These searches gave a total of 275 results, with multiple articles acquired in more of the searches. Twenty-five articles met the inclusion criteria based on the abstract, publication date, and title.

## 4. Conclusions

The development of various preclinical models of thyroid cancer has provided significant advancement in our understanding of the molecular pathogenesis underlying thyroid carcinogenesis and has unveiled opportunities for the development of innovative clinical strategies to manage thyroid cancer.

CDXMs have been utilized to investigate anti-thyroid cancer drugs, focusing on tumor differentiation, vascularization, proliferation, and metastasis. GEMs have been valuable in studying gene alterations, tumorigenesis, identifying anti-thyroid cancer targets, and understanding pharmacological mechanisms. PDXMs, on the other hand, are primarily used for preclinical trials and personalized drug screening.

Signaling pathways such as RAS/RAF/MEK/ERK and PI3K/AKT/mTOR have been extensively studied using mouse models, and gene alterations such as RET/PTC rearrangements and BRAFV600E mutation have been validated as oncogenic in PTC. Additionally, RAS mutations play a role in the development of both PTCs and FTCs. The understanding gained from these studies has contributed to the development and testing of novel anti-thyroid cancer drugs.

Both thyroid cancer cell lines and mouse models have distinct advantages and limitations, and it remains essential to understand the characteristics and challenges of each model and utilize the most appropriate model for specific research goals. Advanced mouse models that closely resemble human thyroid cancer biology are needed to bridge the gap between basic research and clinical studies.

## Figures and Tables

**Table 1:** Summary of mouse models used in differentiated thyroid cancer.

	Implant	Host	Advantages	Drawbacks
CDXMs	Human cancer cell lines	Immunodeficient mice	Easy to generate and monitor	Lack of tumor microenvironment
PDXMs	Patient-derived tissue	Immunodeficient mice	Genetic mutations of human thyroid cancer are preserved	Cannot analyze the effect of immunotherapy
Syngeneic models	Mouse cancer cells	Immunocompetent mice	Intact immune system	Not enough time for the development of tumor-immune cell interaction due to rapid tumor growth
GEMs	None	Immunocompetent, genetically altered mice	Best models to assess tumor-immune cell milieu	High cost

CDXM, cell-derived xenograft model; PDXM, patient-derived xenograft model; GEM, genetically engineered model.

## Data Availability

Not applicable.
